# School Diseases

**Published:** 1906-10-06

**Authors:** J. T. Ainslie Walker

**Affiliations:** Managing Director. For Jeyes' Sanitary Compounds Company, Limited


*To the Editor of* The Hospital.

### SCHOOL DISEASES.

*To the Editor of* The Hospital.

Sir,—With a view to enabling medical officers and principals of schools to minimise the grave risks attending outbreaks of infectious disease, to which young people are especially liable and to which you devoted an important and well-timed leading article in your issue of September 15, may we be allowed to remind you that of the many new departures in sanitary science none has produced more striking results than the rapidly increasing use of efficient disinfectants. In America the practice of disinfecting the floors of schoolrooms has become part of the daily routine; in this connection the Committee on Disinfection of the American Public Health Association, in reviewing the work of the year 1904, state that remarkable results have followed the use of disinfectants when sprayed daily on the floors of schoolrooms, infectious colds and other dust-borne diseases being considerably lessened among the scholars. What applies to schools applies with even greater effect to hospitals. The following particulars will enable the public to apply the cyllin preparations to the greatest advantage in producing a like favourable result in this country. The cyllin disinfectant should be applied daily to all the floors of class-rooms and living rooms (where not carpeted) in the form of a fine spray, and in the flushing of drains, traps, sinks, and urinals. The dilution recommended in all such cases is one part of cyllin to 400 parts of water (1 oz. to 10 quarts of water). The "cyllin sanitary powder" should be used daily in sprinkling all dustbins or other receptacles for dry garbage. The "cyllin soap" (liquid, bar, or soft) may be used for scrubbing purposes. The "cyllin sawdust" is most serviceable for sprinkling carpets before sweeping.

One gallon of cyllin will make 400 gallons of an efficient disinfectant solution, and one gallon of the latter, when properly applied from a spraying machine or fine rose watering-can, is sufficient to sterilise the floor of an average size class-room at a cost so trifling (about one-eighth of a penny) as to be hardly worth considering on the score of expense.

We particularly desire to call the attention of your readers to the fact that the cyllin preparations are sold with a guarantee of efficiency. Cyllin disinfectant is guaranteed to be thirteen times more efficient than pure carbolic acid when tested by the Rideal-Walker method against a vigorous culture of B. typhosus, and "cyllin soaps" are guaranteed to be equal to 50 per cent. carbolic acid, but may be used freely without fear of affecting the hands.

I am, etc.,

J. T. Ainslie Walker, Managing Director.

For Jeyes' Sanitary Compounds Company, Limited.

